# Effect of Huanglian Jiedu Decoction on Thoracic Aorta Gene Expression in Spontaneous Hypertensive Rats

**DOI:** 10.1155/2014/565784

**Published:** 2014-03-13

**Authors:** Gui-Hua Yue, Shao-Yuan Zhuo, Meng Xia, Zhuo Zhang, Yi-Wen Gao, Yuan Luo

**Affiliations:** ^1^Guangxi University of Chinese Medicine, Nanning 530001, China; ^2^Affiliated Ruikang Hospital, Guangxi University of Chinese Medicine, Nanning 530001, China

## Abstract

*Objective*. Hypertension is one of the most common cardiovascular disorders with high mortality. Here we explored the antihypertension effects of Huanglian Jiedu Decoction (HJD) on thoracic aorta gene expression in spontaneous hypertensive rats. *Methods*. A rat model of spontaneous hypertension was used. The gene change profile of thoracic aorta after JHD treatment was assessed by GeneChip(GC) analysis using the Agilent Whole Rat Genome Oligo Microarray. *Results*. Hypertension induced 441 genes upregulated and 417 genes downregulated compared with the normal control group. Treatment of HJD resulted in 76 genes downregulated and 20 genes upregulated. GC data analysis showed that the majority of change genes were involved in immune system process, developmental process, and cell death. *Conclusion*. Hypertension altered expression of many genes that regulate various biological functions. HJD significantly reduced hypertension and altered the gene expression profiles of SHR rats. These changing genes were involved in many cellular functions such as regulating smooth muscle contraction, Ca(2+) homeostasis, and NO pathway. This study provides the potential novel insights into hypertension and antihypertension effects of HJD.

## 1. Introduction

Essential hypertension (primary hypertension or idiopathic hypertension) is the most common type of hypertension, affecting 95% of hypertensive patients. The total number of adults with hypertension in 2000 was 972 million, and the number of adults with hypertension in 2025 was predicted to increase by about 60% to a total of 1.56 billion (29.2% of the world's adult population) [1]. The etiology of primary hypertension is not fully understood. Treatment of hypertension is facing the challenge because effective therapies of hypertension are limited by availability, cost, and adverse effects. Chinese traditional medicine provides a potential option to overcome current challenge of antihypertension therapy. Chinese medicine, a system of ancient medical practice that differs in substance, methodology, and philosophy to modern medicine, plays key role in health maintenance and has become more frequently used.

CM has been used to treat symptoms related to hypertension for more than 2500 years. JHD is one of the effective formulas to combat hypertension in clinic. HJD is an ancient Chinese prescription, first reported in the text* Zh* $\overset{ˇ}{\text{o}}$ *u H*î*u B*è*i J*í* F*ā*ng *(Emergency Standby Remedies) by Gě Hïng (281–341 CE, Jìn dynasty). This formula is commonly used for all types of Fire Toxin obstructing the three burners with high fever, irritability, and dry mouth and throat. Pharmacologic Researches of this formula show that it has antisepticised dephlogisticate and enhances the immune system functions. Our previous studies show that HJD has antihypertension effects via regulating the inflammatory factors and altering NO and SOD expression [2]. Here, this report is to study the pathogenic mechanism of hypertension and its effects on thoracic aorta gene expression.

## 2. Materials and Methods

### 2.1. Animals Models and Treatment

The cohorts of 6-week-old male Spontaneous Hypertensive Rats (SHRs) were randomly placed into HJD group and SHR model group. There are eight rats in each group. All rats were from Beijing Vital River Laboratory Animal Technology Co., Ltd., license Number is SCXK2006-0009, SPF, weight 160 ± 10 g. Eight 6-week-old male normotensive Wistar-Kyoto(WKY) rats were used as the normal control group that were from Experimental Animal Center of Guangxi Medical University, license Number is SCXK2006-0003, SPF, weight 160 ± 10 g.

The animals were housed in a room with 20–24°C 30–40% humidity. Rats were ad lib to food and water. This study was performed following the Guide for the Care and Use of Laboratory Animals of the P. R. China. The protocol was approved by the Committee on the Ethics of Animal Experiments of the Guangxi University of Chinese Medicine.

HJD was consisted of Rhizoma Coptidis,* Phellodendron amurense *Rupr, Radix Scutellariae, and* Gardenia jasminoides *Ellisin the ratio of 3 : 2 : 2 : 3. All the herbs were provided by Ruikang Affiliated Hospital to Guangxi University of Chinese Medicine who bought from Yulin Herb Market in unify. The purity of each component was determined to be above 98% by high-performance liquid chromatography (HPLC) analysis. HPLC-grade reagents, methanol, acetonitrile, and water were obtained from J. T. Baker (Phillipsburg, NJ, USA). These four herbs were dried in 24 h and grinded into coarse powder. The herbs were cooked into concentrated juice (1 mL juice = 2.2 g raw herb). The rats were given HJD juice at 5.4 g/(kg·d) by gavage. The control group was given 0.9% saline with the same volume. All the treatments were administrated by gavage for 6 successive weeks.

### 2.2. Measurement of Blood Pressure

Noninvasive BP of rats was measured using the Nexfin monitor (BMEYE B.V, Amsterdam, and The Netherlands) with the latest implementation of the caudal artery method. Rats were measured for BP at day 0, 7, 14, 28, and 42 after HJD treatment each rat was measured 3 times to obtain average BP value.

### 2.3. RNA Isolation and Microarray Experiment

After the last HJD administration, all rats were sacrificed and then the thoracic aortas were quickly dissected and stored in the freezer tube in −80°C for RNA extraction. Rat tissues were homogenized in Trizol (Invitrogen, Carlsbad, CA), and the total RNA was extracted using chloroform and isopropyl alcohol and purified using the RNeasy Mini Kit and RNase-free DNase Set (Qiagen, Valencia, CA) according to the manufacturers' protocols. The quality of the extracted total RNA was measured using the Agilent 2100 Bioanalyzer (Agilent Technologies, Santa Clara, CA, USA) and the Nano-Chip-protocol before microarray experiments. Gene Chip analysis was performed using the Agilent Whole Rat Genome Oligo Microarray. Labeling and hybridization were performed at Shanghai Biotechnology Corporation.

### 2.4. Data Analysis

Microarray data were analyzed using the SBC Analysis system (: http://sas.ebioservice.com/index.jsp).

Gene expression profiles of SHR were compared to those from WKY or HJD treatment group. The changing was selected by the criteria: *P* < 0.05 and Fold Changes ≥2.

## 3. Results 

### 3.1. HJD Reduced Blood Pressure of SHR Rats

**Figure 1:**
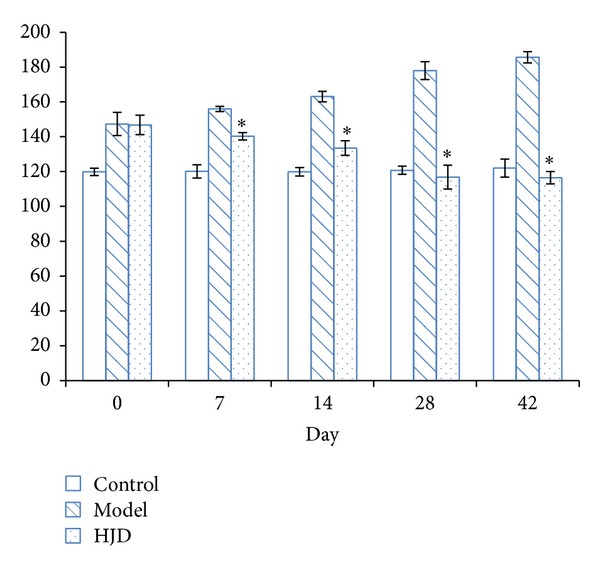
Effects of HJD on blood pressure in rats. BP of rat was measured with mmHg unit. There were 8 rats in each group. *P < 0.05 versus SHR group.

The rat in SHR group had significantly higher blood pressures compared with normal control group before HJD treatment (*P* < 0.05). After one week administration, the BP of HJD group began to decrease than SHR group. After 6 weeks of treatment, the rats in HJD group had almost similar blood pressure to the rats in control group (Figure 1).

### 3.2. Hypertension and HJD Treatment Caused Gene Expression Changes

**Figure 2:**
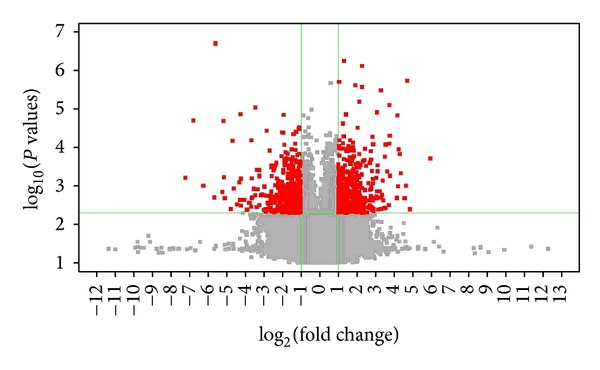
Hypertension induced gene changes compared with normal control rats. Note: model = spontaneous hypertension rats (SHR) model group, and control = normotensive Wistar-Kyoto rats control group.

**Figure 3:**
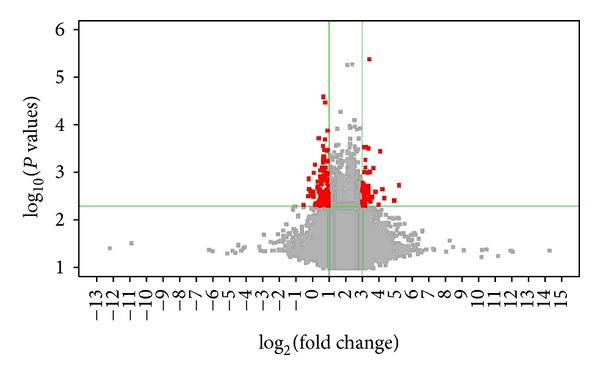
HJD treatment caused gene changes compared with untreated SHR rats versus SHR. Note: model = spontaneous hypertension rats (SHR) model group; medication = HJD treatment group.

Volcano plot that was drawn by *P* value and Fold Change checked by *t*-test showed the significant difference of sample data between chipset groups. (Figures 2 and 3 described by Gene-Spring 11.0: *X*-axis for Log_2_ (Fold Change), *Y*-axis for—Log_10_ (*P* value); *X*-axis parallel line: *P* = 0.05, *Y*-axis parallel line: Fold Change = 2.0; Red Zone: *P* < 0.05; and Fold Change ≥2.0).

Gene microarray analysis showed that there were 858 genes that alter in SHR group compared with normal control groups (Figure 2). There were 417 downregulated (Table 1) and there were 441 upregulated (Table 2).

JHD treatment altered 96 gene expressions pared with untreated SHR group; *P* < 0.05 and Fold Change ≥ 2.0 were identified between the HJD treatment group and the SHR model groups. Of these, 76 genes were downregulated and 20 genes were upregulated (Table 3).

### 3.3. HJD Induced Gene Changes and Their Biological Consequences

The genes that were altered after HJD treatment mainly belong to 12 classifications (Table 4) including genes that regulate immune system, cellular functions, and metabolic and developmental functions. There were 33 genes in the category of immune system development, leukocyte activation, regulation of immune system process, cell activation and proliferation, regulation of immune system process, positive regulation of biological process, and so forth (*P* < 0.05) (Table 5).

Further functional pathway analysis showed that hypertension altered genes were involved in 22 KEGG pathways (Table 6), while HJD altered genes were mainly related to 10 KEGG pathways (*P* < 0.05) (Table 7).

## 4. Discussion and Conclusions

*prehypertension criterion* is systolic pressure 120~139 mmHg (1 mmHg = 0.133 kPa) and/or diastolic pressure 80~89 mmHg. As the important pathogenic risk factor of many cardiovascular and cerebrovascular diseases, prehypertension can affect the structure and function of heart, brain, and liver compared to the ideal blood pressure. During prehypertension, the elasticity of large artery trunks was already obviously damaged, and the relaxation function of left ventricle was slightly damaged, which are the damage symbol of target organs in hypertension [3]. Our previous data showed that SHR arterial elasticity and endothelial functions in SHR rats were impaired in early stage [3]. And the gene array studies coincidences showed that multiple genes in aorta thoracic in young SHR were changed in transcription level compared with normotensive WKY. In particular, expression genes related to smooth muscles shrink or cytoskeletons were significantly increased or decreased. These genes were Chrm3, MRPS2, TRPC4, P2rx4, Slc8a3, Htr5a, Chp, and so forth. The muscarinic cholinergic receptor 3, also known as Chrm3 (FC = 4.343), controls smooth muscle contraction and its stimulation causes secretion of glandular tissue [4]. MRPS2 (FC = 0.081) encodes mitochondrial ribosomal protein S2 [5, 6]. Genes related to Calcium signaling pathway, like TRPC4(FC = 13.374), P2rx2 (FC = 4.075), and P2rx4 (FC = 3.329) expression, increased, whereas Slc8a3 (FC = 0.313), Htr5a (FC = 0.332), and Chp (FC = 0.398) expression decreased, which will cause the imbalance of Ca(2+) in endothelial cells. TRPC4 is well recognized as a prominent Cation channel in the vascular endothelium, which suggested to serve stimulated Ca(2+) entry in a specific endothelial state during the transition from a proliferating to a quiescent phenotype. Thus, TRPC4 may adopt divergent, as yet unappreciated functions in endothelial Ca(2+) homeostasis and emerges as a potential key player in endothelial phenotype switching and tuning of cellular growth factor signaling [7]. P2X receptors are ATP activated channels that allow the passage of ions across cell membranes and participate in regulating renal microvascular function, autoregulation, and hypertension-associated renal vascular injury [8].

The ATP-gated P2X4 ion channel, expressed on endothelial cells and encoded by P2rx4 gene, is crucial to flow-sensitive mechanisms that regulate blood pressure and vascular remodeling. P2rx4 (−/−) mice do not have normal endothelial cell responses to flow, such as influx of Ca(2+) and subsequent production of the potent vasodilator nitric oxide (NO). Additionally, vessel dilation induced by acute increases in blood flow is markedly suppressed in P2rx4 (−/−) mice. Furthermore, P2rx4 (−/−) mice have higher blood pressure and excrete smaller amounts of NO products in their urine than wild-type mice do. Moreover, no adaptive vascular remodeling, that is, a decrease in vessel size in response to a chronic decrease in blood flow, was observed in P2rx4 (−/−) mice [9]. SLC8A3, solute carrier family 8 (sodium/calcium exchanger) members 3, encodes a member of the sodium/calcium exchanger integral membrane protein family. Three mammalian isoforms in family 8 (SLC8A1, SLC8A2, and SLC8A3) and their splice variants are expressed in a tissue-specific manner to mediate Ca(2+) fluxes across the cell membrane and, thus, significantly contribute to maintain Ca(2+) homeostasis in a wide variety of cell types [10]. The gene described in this record is a member of 5-hydroxytryptamine receptor family and encodes a multipass membrane protein that functions as a receptor for 5-hydroxytryptamine and couples to G proteins, negatively influencing cAMP levels via GI and Go [11]. This protein has been shown to function in part through the regulation of intracellular Ca(2+) mobilization [12]. Calcium binding protein p22 (Chp), which belongs to the EF-hand superfamily of Ca(2+)-binding proteins, may act by transducing cellular Ca(2+) signals to downstream effectors in many cell types [13]. It is also known to be an endogenous inhibitor of calcineurin activity [14] and thus inactivates the T cells of the immune system. Prehypertension showed the symptoms of dizziness, headache, anxiety, and irritability [15]. 300 cases of prehypertension were investigated [16] on TCM syndrome on the criterion of* Subhealth of TCM clinical guidelines* which was announced in 2006. The results showed that most of the cases were belonging to patterns of depression of the liver generating pathogenic fire, stagnation of liver qi and spleen deficiency, and interior disturbance of phlegm heat.

HJD were first reported in Gehong' book of* Handbook of Prescription for Emergency*, but it did not give the name of this prescription; however, the first name SLC8A3, solute carrier family 8 (sodium/calcium exchanger) members 3, encodes a member of the sodium/calcium exchanger integral membrane protein family. Three mammalian isoforms in family 8 (SLC8A1, SLC8A2, and SLC8A3) and their splice variants are expressed in a tissue-specific manner to mediate Ca(2+) fluxes across the cell membrane and, thus, significantly contribute to maintain Ca(2+) homeostasis in a wide variety of cell types [10]. The gene described in this record is a member of 5-hydroxytryptamine receptor family and encodes a multipass membrane protein that functions as a receptor for 5-hydroxytryptamine and couples to G proteins, negatively influencing cAMP levels via GI and Go [11]. This protein has been shown to function in part through the regulation of intracellular Ca(2+) mobilization [12]. Calcium binding protein p22 (Chp), which belongs to the EF-hand superfamily of Ca(2+) binding proteins, may act by transducing cellular Ca(2+) signals to downstream effectors in many cell types [13]. It is also known to be an endogenous inhibitor of calcineurin activity [14] and thus inactivates the T cells of the immune system. Prehypertension showed the symptoms of dizziness, headache, anxiety, and irritability [15]. 300 cases of prehypertension were investigated [16] on TCM syndrome on the criterion of* Subhealth of TCM clinical guidelines* which was announced in 2006. The results showed that most of the cases were belonging to patterns of depression of the liver generating pathogenic fire, stagnation of liver qi and spleen deficiency, and interior disturbance of phlegm heat.

HJD were first reported in Gehong' book of* Handbook of Prescription for Emergency*, but it did not give the name of this prescription; however, the first name occurred in Wangtao' book of* Waitaimiyao*. This prescription was made up of Rhizoma Coptidis,* Phellodendron amurense* Rupr, Radix Scutellariae, and* Gardenia jasminoides* Ellis in proportion. Rhizoma Coptidis exerts as the sovereign to purge heart fire and middle energizers fire;* Phellodendron amurense* Rupr works as the minister to clear away lung heat and the upper energizers fire. Radix Scutellariae clears away the lower energizers fire and* Gardenia jasminoides* Ellis clears away the triple energizers fire. They work together to remove the toxin. HJD have played more and more important role in cardiovascular diseases that have already used HJD in treatment of hypertension in clinic for many years.

Hypertension induces endothelial dysfunction resulting in impairment of endothelium-dependent vasodilatation and widespread abnormalities in endothelial integrity and homeostasis [17]. Endothelial dysfunction exhibits several pathological conditions, including altered anticoagulant and anti-inflammatory properties of the endothelium, impaired modulation of vascular growth, and dysregulation of vascular remodeling. The major cause of impairment of endothelium-dependent vasorelaxation is loss of nitric oxide (NO) bioactivity in the vessel wall [18].

Our previous studies showed that' spontaneous hypertension rats' blood pressure increases at six-week-old age, and the tail-arterial-systolic-pressure increase at 12-weeksof age. Hypertension decreases plasma NO and SOD, and increase of MDA and Von Wille brand factor (vWF) at the 12 weeks of age. HJD treatment significantly reversed hypertension-induced NO and SOD [2].

HJD treatment altered many genes related to cell response processes, hyperplasia, and immune response, such as IL1b(FC = 2.519), Klrk1(FC = 2.001), Snca(FC = 2.665), and Gch1(FC = 2.164). It has been reported that interleukin (IL)-1 beta induces the synthesis of both nitric oxide (NO) and prostaglandin (PG)E2 in cultures of dispersed ovarian cells and exerts cytotoxic effects on these cells [19, 20]. Furthermore, IL-1 beta can interact with NO and PGE2 during steroid genesis [21]. Klrk1 is involved in positive regulation of nitrogen compound metabolic process (*Gene Ontology*:* 0051173*). Alpha-synuclein (ASN), encoded by Snca gene, is a small soluble acidic protein and plays an important role in the regulation of synaptic nerve terminal function. It has been shown that ASN stimulated NOS activity and NO release in the rat brain [22]. GTP cyclohydrolase 1 (GCH1) is the first and rate-limiting enzyme in the synthesis of tetrahydrobiopterin (BH4), an essential cofactor for tyrosine hydroxylase (TH) and NOS in the catecholamine and NO production pathways, respectively, thus playing a crucial role in the sympathoadrenal and endogenous nitro vasodilator systems [23].

According to the GO analyses, compared with normotensive WKY, the biological procedure which SHR differential expression genes attended mainly focused on 6 items of secondary classification, such as multicellular organismal process, developmental process, multiorganism process, response to stimulus, anatomical structure formation, and regulation of biological process, mainly involving 12 items of third classification, for example, ossification, response to other organism, regulation of multicellular organismal process, regulation of developmental process, interspecies interaction between organisms, tissue remodeling, response to biotic stimulus, and so froth (*P* < 0.05) (Table 5).

HJD can upregulate NO expression in the blood plasma [4]. Here HJD also altered the NO pathway related genes including IL1b, Klrk1, Snca, and Gch1. Taking together, these findings suggest that HJD may reduce hypertension by regulating NO pathways.

In conclusion, HJD can significantly reduce hypertension and alter the gene profiles in aorta thoracic in rats. These results provide the potential gene changes and pathways underlying hypertension and antihypertension effects of HJD in treatment of hypertension.

## Figures and Tables

**Table 1:** Downregulated genes in hypertension rats.

Gene	Abbr.	Gene Id	Chro.	FC	*P*
DRABUB01 rat DRG library Rattus norvegicus cDNA clone DRABUB01 5′	—	NA	9	0.097	3.86*E* − 03
Similar to RIKEN cDNA 1700001F09 (LOC289957)	—	NA	15	0.092	9.47*E* − 05
Mitochondrial ribosomal protein S2	Mrps2	362094	3	0.081	6.49*E* − 03
Q99LR9_MOUSE (Q99LR9) Riok3 protein (fragment), partial (13%)	—	NA	18	0.079	6.79*E* − 04
AGENCOURT_26625193 NIH_MGC_253 Rattus norvegicus cDNA clone IMAGE:7304073 5′	—	NA	5	0.054	6.58*E* − 03
Similar to 60S ribosomal protein L19 (LOC316856)	—	NA	Un	0.053	1.40*E* − 04
Solute carrier family 4 (anion exchanger), member 1	Slc4a1	24779	10	0.051	8.24*E* − 03
Cell division cycle 25 homolog A (S. pombe)	Cdc25a	171102	8	0.039	6.88*E* − 04
Synuclein, alpha (non-A4 component of amyloid precursor)	Snca	29219	4	0.029	6.00*E* − 03
Unknown	—	NA	7	0.028	2.12*E* − 04
Coproporphyrinogen oxidase	Cpox	304024	11	0.021	2.11*E* − 06
AGENCOURT_70342463 NIH_MGC_368 Rattus norvegicus cDNA clone IMAGE:8376762 5′	—	NA	1	0.013	9.98*E* − 03
Q8FIR6_ECOL6 (Q8FIR6) cytochrome b561 homolog 2, partial (5%)	—	NA	9	0.009	2.05*E* − 04
UI-R-C3-sf-g-04-0-UI	—	NA	1	0.007	6.28*E* − 03

Note. Abbr.: gene abbreviation, Chro.: chromosome, Gene Id represents the identity (ID) of the gene in GenBank; FC = SHR model group gene expression level/normotensive WKY control group expression level. “—/NA/Un” indicates unknown.

**Table 2:** Upregulated genes (model versus control).

Gene	Abbr.	Gene Id	Chro.	FC	*P*
Bone gamma-carboxyglutamate (gla) protein	Bglap	25295	2	61.402	1.96*E* − 03
Uncharacterized protein	—	NA	2	25.856	1.96*E* − 05
Unknown	—	NA	13	24.949	9.99*E* − 03
Cellular retinoic acid binding protein 2	Crabp2	29563	2	20.657	4.73*E* − 03
Major histocompatibility complex, assembled from 40 BACs, strain Brown Norway (BN/ssNHsd), RT1n haplotype; segment 2/11	—	NA	20	19.648	1.52*E* − 03
V-set domain containing T cell activation inhibitor 1	Vtcn1	295322	2	18.951	1.15*E* − 03
Interferon-induced protein with tetratricopeptide repeats 1	Ifit1	56824	1	17.991	1.51*E* − 04
Unknown	—	NA	10	13.875	5.54*E* − 03
Uncharacterized protein	—	NA	1	13.621	5.06*E* − 04
Similar to SMAD-interacting zinc finger protein 2	LOC679126	679126		13.572	8.21*E* − 05
Transient receptor potential cation channel, subfamily C, member 4	Trpc4	84494	2	13.374	8.20*E* − 04
Q3X0T1_9ACTN (Q3X0T1) 60 kDa innermembrane protein, partial (6%)	—	NA	19	12.417	4.35*E* − 03
CJ032_MOUSE (Q9CRC6) protein C10orf32 homolog, partial (15%)	—	NA	1	11.726	8.63*E* − 04
TL0ACA45YL24	—	NA	X	11.407	1.80*E* − 03

Note. Abbr.: gene abbreviation, Chro.: chromosome, Gene Id represents the identity (ID) of the gene in GenBank; FC = SHR model group gene expression level/normotensive WKY control group expression level. “—/NA/Un” indicates unknown.

**Table 3:** The upregulated and downregulated genes after the treatment of HLJDD.

Gene	Abbr.	Gene Id	Chro.	FC	*P*
Solute carrier family 4 (anion exchanger), member 1	Slc4a1	24779	10	3.871	6.74*E* − 03
Protein phosphatase 1, regulatory subunit 3D	Ppp1r3d	689995	3	2.566	2.78*E* − 03
Ribonucleotide reductase M2	Rrm2	362720	6	2.551	6.30*E* − 03
pim-1 oncogene	Pim1	24649	20	2.529	5.12*E* − 03
Spectrin, beta, erythrocytic	Sptb	314251	6	2.470	4.66*E* − 03
Transmembrane and coiled-coil domain family 2	Tmcc2	305095	13	2.444	3.38*E* − 03
Unknown	—	NA	3	2.264	7.87*E* − 03
UI-R-C4-alc-g-09-0-UI	—	NA	Un	0.235	3.60*E* − 03
UI-R-BJ2-bql-b-07-0-UI	—	NA	13	0.385	3.14*E* − 03
Potassium voltage-gated channel, subfamily H (eag-related), member 1	Kcnh1	65198	13	0.393	9.98*E* − 03
Nuclear receptor subfamily 4 group A member 3	Nr4a3	58853	5	0.456	9.87*E* − 03

Note. Abbr.: gene abbreviation, Chro.: chromosome, Gene Id represents the identity (ID) of the gene in GenBank; FC = HLJDT treatment group/SHR model group gene expression level. “—/NA/Un” indicates unknown.

**Table 4:** Functional catalogers of hypertension-induced gene change profiles.

GO Id	Term	Hit genes	*P*
GO:0032501	Multicellular organismal process		
GO:0051239	Regulation of multicellular organismal process	56	0.0033
GO:0048771	Tissue remodeling	14	0.0068
GO:0001816	Cytokine production	15	0.0243
GO:0003008	System process	77	0.0485
GO:0032502	Developmental process		
GO:0001503	Ossification	18	0.0005
GO:0050793	Regulation of developmental process	66	0.0045
GO:0051704	Multiorganism process		
GO:0051707	Response to other organism	23	0.0026
GO:0044419	Interspecies interaction between organisms	7	0.0059
GO:0050896	Response to stimulus		
GO:0009607	Response to biotic stimulus	24	0.0074
GO:0042221	Response to chemical stimulus	97	0.0214
GO:0010926	Anatomical structure formation		
GO:0022607	Cellular component assembly	41	0.042
GO:0050789	Regulation of biological process		
GO:0051239	Regulation of multicellular organismal process	56	0.0033
GO:0050793	Regulation of developmental process	66	0.0045
GO:0048518	Positive regulation of biological process	86	0.0361

**Table 5:** Functional category of HJD-induced gene change profiles.

GO Id	Term	Hit genes	*P*
GO:0002376	Immune system process		
GO:0002520	Immune system development	10	0.0001
GO:0045321	Leukocyte activation	12	0.0001
GO:0002682	Regulation of immune system process	12	0.0001
GO:0006955	Immune response	10	0.0003
GO:0002684	Positive regulation of immune system process	7	0.0006
GO:0045058	T cell selection	2	0.0046
GO:0002253	Activation of immune response	3	0.026
GO:0009987	Cellular process		
GO:0001775	Cell activation	13	0.0001
GO:0008283	Cell proliferation	15	0.0001
GO:0048522	Positive regulation of cellular process	22	0.0002
GO:0008219	Cell death	13	0.0016
GO:0048523	Negative regulation of cellular process	16	0.0086
GO:0016043	Cellular component organization	19	0.0181
GO:0007049	Cell cycle	7	0.0453
GO:0008152	Metabolic process		
GO:0009058	Biosynthetic process	28	0.0005
GO:0009893	Positive regulation of metabolic process	11	0.0361
GO:0006807	Nitrogen compound metabolic process	5	0.0413
GO:0019748	Secondary metabolic process	2	0.0423
GO:0032502	Developmental process		
GO:0050793	Regulation of developmental process	17	0.0008
GO:0051093	Negative regulation of developmental process	10	0.0026
GO:0048856	Anatomical structure development	21	0.0086
GO:0009790	Embryonic development	8	0.0154
GO:0007275	Multicellular organismal development	20	0.0249
GO:0001503	Ossification	4	0.0251
GO:0009653	Anatomical structure morphogenesis	12	0.0409
GO:0016265	Death		
GO:0008219	Cell death	13	0.0016
GO:0032501	Multicellular organismal process		
GO:0001816	Cytokine production	7	0.0003
GO:0051240	Positive regulation of multicellular organismal process	7	0.0017
GO:0051239	Regulation of multicellular organismal process	14	0.0021
GO:0007275	Multicellular organismal development	20	0.0249
GO:0050896	Response to stimulus		
GO:0006955	Immune response	10	0.0003
GO:0009605	Response to external stimulus	11	0.0084
GO:0009607	Response to biotic stimulus	6	0.0201
GO:0048584	Positive regulation of response to stimulus	5	0.0209
GO:0048585	Negative regulation of response to stimulus	3	0.0445
GO:0050789	Regulation of biological process		
GO:0002682	Regulation of immune system process	12	0.0001
GO:0048518	Positive regulation of biological process	24	0.0001
GO:0050793	Regulation of developmental process	17	0.0008
GO:0051239	Regulation of multicellular organismal process	14	0.0021
GO:0048519	Negative regulation of biological process	18	0.0045
GO:0032844	Regulation of homeostatic process	3	0.045
GO:0048518	Positive regulation of biological process	24	0.0001
GO:0048519	Negative regulation of biological process	18	0.0045
GO:0010926	Anatomical structure formation	12	0.0254

**Table 6:** Hypertension altered genes in 22 KEGG pathways.

KEGG pathway	Hit genes	*P*
Olfactory transduction	29	0
Neuroactive ligand-receptor interaction	13	0.0002
Cytokine-cytokine receptor interaction	10	0.0009
Focal adhesion	9	0.0009
Tight junction	6	0.0061
RIG-I-like receptor signaling pathway	4	0.0081
Ether lipid metabolism	3	0.0083
Calcium signaling pathway	7	0.0086
Leishmania infection	4	0.0089
Amyotrophic lateral sclerosis (ALS)	4	0.0113
Arrhythmogenic right ventricular cardiomyopathy (ARVC)	4	0.014
ABC transporters	3	0.0171
Aldosterone-regulated sodium reabsorption	3	0.0213
Hypertrophic cardiomyopathy (HCM)	4	0.0231
Dilated cardiomyopathy	4	0.0247
Glycosphingolipid biosynthesis—ganglio series	2	0.0265
Glutathione metabolism	3	0.0284
Apoptosis	4	0.032
Wnt signaling pathway	5	0.0324
Natural killer cell mediated cytotoxicity	5	0.0339
Maturity onset diabetes of the young	2	0.0415
MAPK signaling pathway	7	0.047

**Table 7:** HJD altered genes in 10 KEGG pathways.

KEGG pathway	Hit genes	*P*
Hematopoietic cell lineage	3	0.0004
Metabolic pathways	8	0.0016
Natural killer cell mediated cytotoxicity	3	0.0027
NOD-like receptor signaling pathway	2	0.0066
Cytokine-cytokine receptor interaction	3	0.0105
Pyrimidine metabolism	2	0.0148
T cell receptor signaling pathway	2	0.0192
Axon guidance	2	0.0254
Jak-STAT signaling pathway	2	0.0308
Purine metabolism	2	0.0379
